# Immune evasion, dysregulation, and emerging immunotherapies for invasive fungal infections in the immunocompromised host

**DOI:** 10.3389/fimmu.2026.1788786

**Published:** 2026-04-01

**Authors:** Haiying Liu, Chunyan Wang, Weijuan Huang, Shuhua Hu, Congfu Huang

**Affiliations:** 1 Department of Pediatrics, Shenzhen Maternity and Child Healthcare Hospital, Women and Children’s Medical Center, Southern Medical University, Shenzhen, Guangdong, China; 2 Department of Pediatrics, Shenzhen Fourth People’s Hospital(Sami Medical Center), Shenzhen, Guangdong, China; 3 Department of Pediatrics, Longgang District Maternity & Child Healthcare Hospital of Shenzhen City (Longgang Maternity and Child Institute of Shantou University Medical College), Shenzhen, Guangdong, China

**Keywords:** CAR-T cells, immune checkpoint inhibitors, immune dysregulation, immune evasion, immunocompromised host, immunotherapy, invasive fungal infections, precision medicine

## Abstract

Invasive fungal infections (IFIs) represent a persistent challenge to global health, particularly in immunocompromised individuals, among whom they remain a leading cause of morbidity and mortality. Conventional antifungal regimens face growing limitations due to the emergence of drug resistance, drug-associated toxicities, and suboptimal efficacy. This review synthesizes advances from 2015 to 2025 in understanding the dynamic interactions between major fungal pathogens—*Candida albicans*, *Aspergillus fumigatus*, and *Cryptococcus neoformans*—and the host immune system, emphasizing both conserved and species-specific immune evasion mechanisms. These sophisticated strategies include surface antigen masking, biofilm formation, metabolic adaptation, and the secretion of immunomodulatory effectors, which collectively facilitate fungal persistence and dissemination. In the immunocompromised host, these evasion tactics are compounded by profound immune dysregulation, characterized by aberrant activation of the NOD-, LRR-, and pyrin domain-containing protein 3 (NLRP3) inflammasome, deficiencies in T helper type 1 (Th1) and type 17 (Th17) responses, T-cell exhaustion, and upregulation of inhibitory checkpoints such as programmed cell death protein 1 (PD-1). These disruptions not only compromise pathogen clearance but also exacerbate immunopathology and tissue damage. Advancing beyond conventional antifungal paradigms, we highlight a dual-track therapeutic framework that integrates direct antifungal activity with tailored immunomodulation. Promising therapeutic avenues encompass immune checkpoint inhibitors, cytokine therapies, prophylactic and therapeutic vaccines, monoclonal antibodies, and adoptive cellular therapies such as chimeric antigen receptor T (CAR-T) cells. We further discuss the integration of multi-omics profiling and personalized immune monitoring to guide precision immunotherapy. Finally, we outline critical translational challenges and future directions aimed at improving clinical outcomes for immunocompromised patients afflicted with IFIs.

## Graphical Abstract

**Figure:**
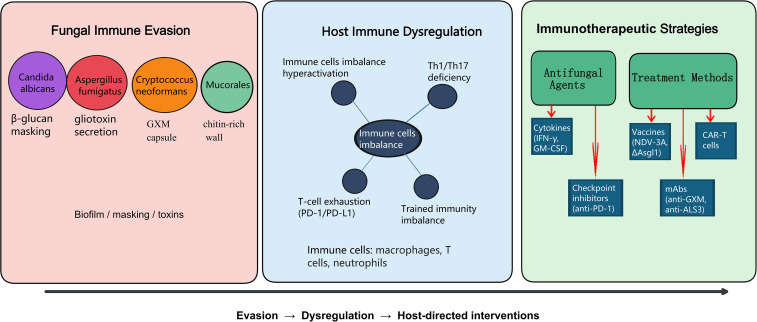
Immune evasion, dysregulation, and immunotherapy for invasive fungal infections in immunocompromised hosts. This graphical abstract summarizes the interplay between fungal immune evasion, host immune dysregulation, and emerging immunotherapeutic strategies in immunocompromised patients with invasive fungal infections (IFIs). Left panel, Major fungal pathogens—*C. albicans*, *A. fumigatus*, and *C. neoformans*—employ key immune evasion mechanisms, including surface antigen masking (e.g., β-glucan concealment), biofilm formation, toxin secretion (e.g., gliotoxin), and polysaccharide capsule deployment (e.g., GXM). Central panel, In immunocompromised hosts (e.g., HSCT recipients, advanced HIV/AIDS, autoimmune patients), these evasion tactics drive profound immune dysregulation. This includes aberrant activation of the NLRP3 (NOD-, LRR-, and pyrin domain-containing protein 3) inflammasome, deficiencies in T helper type 1 (Th1) and type 17 (Th17) responses, T-cell exhaustion (e.g., upregulation of programmed cell death protein 1 PD−1 and its ligand PD-L1), and disrupted trained immunity, collectively impairing pathogen clearance while exacerbating tissue injury. Right panel, A dual-track therapeutic framework integrates direct antifungal agents with tailored immunomodulation. Promising strategies include immune checkpoint inhibitors (e.g., anti-PD-1), cytokine therapies (e.g., IFN-γ, GM-CSF), monoclonal antibodies, vaccines (e.g., NDV-3A, Δsgl1), and adoptive cellular therapies (e.g., CAR-T cells). Bottom arrow: The flow from “Evasion → Dysregulation → Therapy” underscores the shift toward a host–pathogen integrated therapeutic paradigm, aiming to restore immune balance and improve clinical outcomes in high-risk patients.

## 1 Introduction

Invasive fungal infections (IFIs) constitute a mounting and life-threatening clinical challenge, particularly among immunocompromised individuals. This high-risk population encompasses recipients of hematopoietic stem cell or solid organ transplants, patients undergoing cytotoxic chemotherapy, individuals with advanced HIV/AIDS, and those on long-term immunosuppressive therapies for autoimmune or inflammatory conditions (1, 2). The severity of this issue is underscored by epidemiological data: IFIs are associated with mortality rates ranging from 40% to 90% in specific high-risk cohorts, such as neutropenic patients with invasive aspergillosis or liver transplant recipients (3). The global burden of fungal disease continues to escalate, a trend driven by the expanding use of immunomodulatory agents, broad-spectrum antibiotics, and invasive medical procedures (1, 2, 4). Clinically prevalent IFIs, including invasive candidiasis, aspergillosis, cryptococcosis, and mucormycosis, contribute significantly to extended hospitalizations, substantial healthcare expenditures, and poor patient outcomes, highlighting an urgent need for more effective therapeutic interventions.

Conventional antifungal drugs, while foundational to management, are hampered by significant limitations. The emergence of multidrug-resistant strains, dose-limiting toxicities, and their often suboptimal efficacy in profoundly immunocompromised hosts increasingly constrain their clinical utility (5, 6). This therapeutic impasse underscores the necessity of moving beyond purely pathogen-centric approaches and toward a deeper understanding of the dynamic interplay between fungal pathogens and the host immune system—a nexus that holds promise for revealing novel immunomodulatory targets.

The principal fungal pathogens responsible for invasive human disease—*Candida albicans, Aspergillus fumigatus, Cryptococcus neoformans/gattii* species complex, and members of the Mucorales order—possess remarkable adaptive abilities to evade or subvert host defenses through an arsenal of conserved and species-specific strategies (7, 8). Notable examples include cell wall remodeling and biofilm formation by *C. albicans* (9, 10), the antiphagocytic polysaccharide capsule of *C. neoformans* (11), and the secretion of immunosuppressive toxins such as gliotoxin by *A. fumigatus* (12, 13). These evasion mechanisms are particularly effective in the context of host immune compromise, where defects in neutrophil function, T helper type 1 (Th1) and type 17 (Th17) cell responses, and overall T cell effector function (14–16) collectively create a permissive environment for fungal dissemination and persistence.

Despite considerable progress, critical knowledge gaps remain. First, what are the precise molecular mechanisms underpinning fungal immune evasion, and how do these strategies diverge between laboratory-adapted strains and clinically relevant isolates? Second, in immunosuppressed hosts, how do imbalances in immune responses—spanning from hyperinflammation to functional paralysis—shift from protective to pathogenic, thereby driving tissue damage and infection progression? Third, how can mechanistic insights into host-fungal interactions be effectively translated into immunotherapeutic strategies that synergize with conventional antifungals?

This review systematically synthesizes cutting−edge evidence (2015–2025) on the interplay between major fungal pathogens and the host immune system in immunocompromised settings. We critically elucidate fungal immune−evasion tactics, delineate mechanisms of host immune dysregulation, and highlight emerging therapeutic targets. Furthermore, we appraise preclinical and early−clinical evidence for various immunomodulatory strategies and propose an integrated “direct antifungal + host−directed immunomodulation” framework. A graphical abstract is provided to visually conceptualize the central themes of this review: the dynamic interplay between fungal immune evasion strategies, host immune dysregulation in immunocompromised states, and the emerging dual-track therapeutic framework that integrates direct antifungal agents with precision immunomodulation. Finally, we outline translational challenges and future directions, emphasizing the role of multi−omics profiling and personalized immune monitoring in improving outcomes for immunocompromised patients with IFIs.

In synthesizing therapeutic strategies (Section 4) and outlining future directions (Sections 5), particular emphasis was placed on translational challenges unique to immunocompromised populations. This includes discussions on safety (e.g., risk of immune-related adverse events, exacerbation of graft-versus-host disease), efficacy limitations in profound immunodeficiency, and the empirical nature of treatment timing and sequencing. The review advocates for a precision medicine framework, discussing the requisite tools—such as single-cell RNA sequencing, dynamic immune phenotyping, and pathogen genomics—and the need for biomarker-driven clinical trial designs to implement personalized immunotherapy successfully.

## 2 Fungal immune evasion: from molecular mechanisms to therapeutic vulnerabilities

Fungal pathogens utilize a diverse and sophisticated arsenal of evolutionarily conserved and species-specific strategies to subvert, evade, and manipulate host immune surveillance, thereby enabling persistent colonization and dissemination—a vulnerability that is particularly pronounced in immunocompromised hosts. Emerging evidence increasingly posits these mechanisms not as passive defenses, but as active and dynamic adaptations, shaped by persistent host-pathogen co-evolutionary pressures (7, 8). This section, therefore, aims to transcend a mere descriptive catalog of individual findings by synthesizing accumulated evidence, critically evaluating conserved themes, and delineating key distinctions across major invasive fungal species, including *C. albicans, A. fumigatus, C. neoformans*, and members of the *Mucorales* order.

### 2.1 Surface antigen variation and molecular disguise

A principal immune evasion strategy employed by invasive fungal pathogens involves the dynamic modification and concealment of cell wall pathogen-associated molecular patterns (PAMPs), notably β-glucans, to subvert recognition by host pattern recognition receptors (PRRs) (7). This evolutionarily conserved tactic is exemplified by *C. albicans*, which extensively remodels its cell surface through O- and N-linked glycosylation. This process masks immunostimulatory β-(1,3)-glucan, thereby impairing its recognition by the pattern recognition receptor Dectin-1 on host immune cells (7–9). However, the *in vivo* relevance and temporal regulation of this glycosylation-based masking during disseminated human infection remain incompletely defined. Mechanistic studies, primarily using laboratory strains, suggest that surface glycosylation masks β-glucans (7, 9). Notably, studies on clinical isolates of related fungi, such as *Candida glabrata*, reveal heightened phenotypic plasticity and altered cell wall protein expression, which could further impede immune recognition (17, 18). The conidial surface protein CcpA has been identified as essential for virulence, likely contributing to immune evasion by masking other surface epitopes or modulating host interactions (19).

Conversely, pathogen-specific strategies involve unique structural features. *C. neoformans* is encapsulated by a thick, viscous polysaccharide capsule composed primarily of glucuronoxylomannan (GXM). This capsule acts as a formidable physical barrier, directly inhibiting phagocytosis by macrophages and neutrophils (11). Beyond its steric role, GXM exhibits potent immunomodulatory properties. It can bind host complement regulatory proteins such as Factor H, thereby inhibiting complement activation and opsonization, a clever co-option of host regulatory mechanisms to diminish immune clearance (20, 21). The cell walls of Members of the Mucorales order, such as those of Mucor and Rhizopus species, are characterized by high chitin and chitosan content. This distinct structural composition confers resistance to degradation by host enzymes and may also influence immune recognition differently compared to the glucan-mannan layers of *Candida* and *Aspergillus* (22). A comparative summary of the primary immune evasion strategies and key molecular effectors for these major fungal pathogens is provided in **Supplementary Table S1**. This table highlights both conserved and species-specific mechanisms, underscoring the need for pathogen-tailored immunotherapeutic approaches. These surface-based camouflage strategies, both conserved (e.g., β-glucan masking) and species-specific (e.g., the *C. neoformans* GXM capsule), highlight critical therapeutic vulnerabilities. For instance, agents that unmask β-glucan, such as echinocandins, could enhance immune recognition across multiple fungal species, while targeting unique structures like GXM with specific monoclonal antibodies offers a pathway for precision intervention (7, 12, 20).

### 2.2 Biofilm formation and immunological shielding

Biofilm formation is a critical contributor to antifungal resistance and immune evasion; however, the mechanistic evidence remains largely descriptive. Most studies rely on *in vitro* or catheter-associated models, which may not fully recapitulate the complexity of mucosal or tissue-embedded biofilms *in vivo*. Moreover, interspecies differences in biofilm matrix composition (e.g., *C. albicans vs. A. fumigatus*) suggest that therapeutic strategies targeting biofilms may require pathogen-specific approaches, a nuance often overlooked in broad discussions of fungal biofilms. Pathogens such as *C. albicans* and *A. fumigatus* can adhere to both biotic (e.g., *mucosal surfaces*) and abiotic (e.g., medical devices) surfaces, forming structured microbial communities encased in a self-produced extracellular matrix (ECM) (23, 24). The ECM, a complex mixture of polysaccharides (β-glucans, mannans), extracellular DNA, and proteins, creates a robust physical and chemical diffusion barrier. This barrier significantly impedes the infiltration of effector immune cells, such as neutrophils and macrophages, and restricts the penetration of antifungal agents, thereby establishing a sanctuary for persistent infection.

The compositional differences in the ECM significantly influence immune cell infiltration and antifungal penetration. For example, *C. albicans* biofilms are rich in β-glucans and extracellular DNA, which hinder immune cell penetration (23). In contrast, *A. fumigatus* biofilms are characterized by a matrix rich in galactomannan and hydrophobic proteins, which contribute to antifungal resistance and immune evasion (24, 25). These distinctions highlight the need for pathogen-tailored therapeutic interventions.

The biofilm mode of growth is not a passive aggregation but a highly regulated developmental process. Fungi within biofilms employ quorum-sensing mechanisms to coordinate population-wide gene expression, leading to the upregulated production of virulence and adherence factors specifically adapted for the biofilm lifestyle. In *C. albicans*, adhesins such as Hwp1 and Als3 are crucial for stabilizing biofilm architecture and mediating firm attachment to host substrates. Their finely tuned expression within the biofilm further solidifies the structure and enhances resistance to immune-mediated dispersal (18, 26). The phenotypic heterogeneity and altered metabolic state of biofilm-embedded cells additionally contribute to their recalcitrance to host defenses.

The structural and immunological shielding provided by biofilms represents not only a key evasion mechanism but also a target for combinatorial therapy. Disrupting the ECM with enzymes like glycoside hydrolases, or enhancing neutrophil infiltration with cytokines such as GM-CSF, could re-sensitize biofilm-embedded fungi to both immune effectors and conventional antifungals (23, 25). Furthermore, antibodies targeting biofilm matrix components (e.g., β-glucan) could disrupt biofilm architecture, increasing fungal susceptibility to immune-mediated clearance (23, 24).

### 2.3 Metabolic adaptation and nutrient competition

Successful colonization and invasion by fungal pathogens necessitate adaptation to the nutrient-limited and hostile host microenvironment, a process in which metabolic flexibility plays a direct and crucial role in immune evasion. The mold *A. fumigatus* exemplifies this through its sophisticated high-affinity iron acquisition systems. By secreting siderophores (e.g., ferricrocin, triacetylfusarinine C), the fungus scavenges iron from the host, a strategy that not only fulfills an essential nutritional requirement but also impairs iron-dependent host defenses, such as the formation of neutrophil extracellular traps (27, 28). This competition for micronutrients thus constitutes an active virulence mechanism. Furthermore, fungal pathogens remodel their core metabolism to withstand host-imposed stresses. Within hypoxic necrotic tissue cores, they upregulate glycolytic pathways and enhance antioxidant defenses (e.g., glutathione systems) to counteract phagocyte-derived reactive oxygen species (ROS) (29). Beyond mere survival, fungi can actively manipulate host immunity through metabolic byproducts. For instance, *C. albicans* secretes acetate during hyphal growth, a metabolite that can influence host macrophage polarization toward an anti-inflammatory M2 phenotype, thereby suppressing the pro-inflammatory, fungicidal M1 state and facilitating fungal persistence and tissue invasion (30). These metabolic adaptations represent not only survival strategies but active virulence mechanisms that can be therapeutically exploited. Targeting fungal iron acquisition with chelators like deferasirox, or counteracting immunometabolic reprogramming with M1-polarizing cytokines (e.g., IFN-γ), represents a promising avenue to impair fungal fitness while simultaneously bolstering host defenses (27, 30).

### 2.4 Secretion of immunomodulatory effector molecules

Fungi actively subvert immune function by secreting a repertoire of effector molecules that directly target and disarm host defenses. A prominent example is gliotoxin, a potent mycotoxin produced by A. fumigatus, which exhibits immunosuppressive properties by inhibiting macrophage phagocytosis. This is achieved through the disruption of phosphatidylinositol 3,4,5-trisphosphate (PIP3) homeostasis and key signaling pathways such as PI3K, leading to defective phagosome maturation and even induction of apoptosis (12, 13). *C. albicans* expresses a family of aspartyl proteases (Saps) that play dual roles in nutrient acquisition and immune evasion. Certain Sap isoenzymes cleave complement components, including C3b and C5a, thereby dampening opsonization and chemotaxis (31). The upregulation of Sap expression in clinical isolates of Candida parapsilosis from invasive infections, which correlates with enhanced immune evasion capacity, suggests a conserved role for Saps across pathogenic Candida species (32). Another critical *C. albicans* virulence factor is Candidalysin, a peptide toxin derived from the Ece1 protein. Candidalysin directly damages epithelial barriers and, notably, serves as a potent activator of the NLRP3 inflammasome, triggering IL-1β release (33, 34). This exemplifies a sophisticated evasion strategy: while epithelial damage facilitates invasion, the concomitant activation of a key inflammatory pathway may paradoxically contribute to immune dysregulation in susceptible hosts, a concept explored in the next section. The direct targeting of immune cells by secreted effectors like gliotoxin and the paradoxical activation of inflammatory pathways by Candidalysin underscore the dual nature of these molecules. Consequently, neutralizing these effectors with specific antibodies or small-molecule inhibitors could simultaneously preserve immune function and mitigate damaging hyperinflammation (12, 33).

Beyond these well-characterized secreted molecules, emerging evidence highlights the role of extracellular vesicles (EVs) in fungal immune modulation. *C. neoformans* releases EVs carrying glucuronoxylomannan (GXM) and other virulence factors, which can modulate macrophage responses and promote fungal persistence (35, 36). Similarly, *C. albicans* EVs transport immunomodulatory proteins that suppress host inflammatory pathways (36). Intriguingly, these EV components have also been exploited as antigen platforms for vaccine development, illustrating the dual role of fungal-derived structures in both pathogenesis and immunoprophylaxis (35, 37).

**Figure 1:**
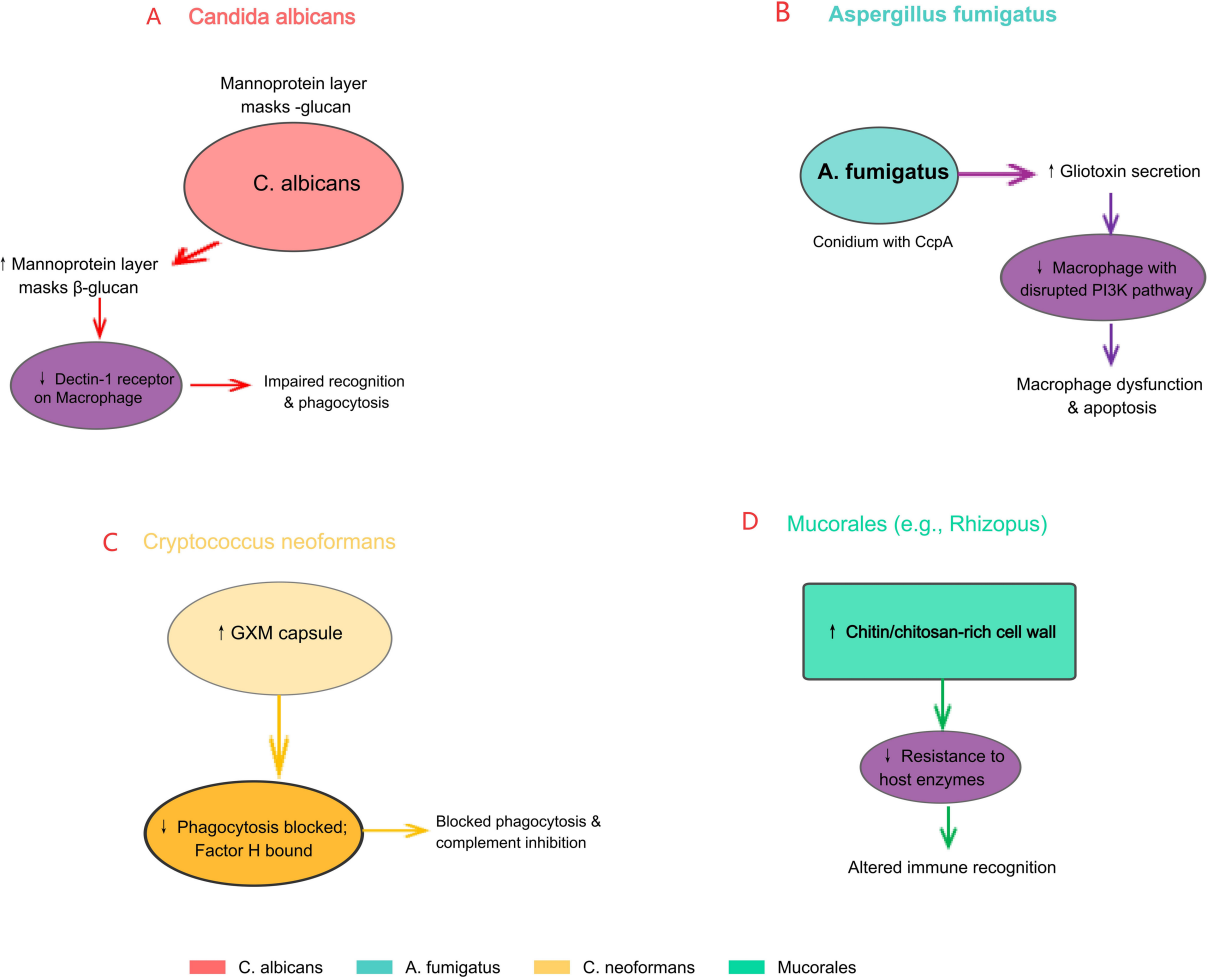
Key immune evasion mechanisms employed by major invasive fungal pathogens. **(A)** *C. albicans* masks β-glucan under a glycosylated mannoprotein layer, impairing Dectin-1 recognition. **(B)** *A. fumigatus* secretes gliotoxin to disrupt macrophage phagocytosis and induce apoptosis. **(C)** *C. neoformans* utilizes a thick GXM capsule to block phagocytosis and scavenge complement factors. **(D)** Members of the Mucorales order exhibit chitin-rich cell walls conferring enzymatic resistance. These mechanisms highlight potential targets for immunotherapy, such as β-glucan unmasking or pathogen-specific monoclonal antibodies.

To provide a consolidated overview of these complex host-pathogen interactions, **Figure 1** schematically illustrates the key immune evasion strategies employed by major fungal pathogens, including surface antigen masking, biofilm formation, toxin secretion, and polysaccharide capsule deployment. **Table 1** summarizes the major conserved and species-specific immune evasion mechanisms, their primary immunological consequences, and their potential therapeutic implications. This framework directly informs the dual-track therapeutic approach discussed in subsequent sections. For an extended, pathogen-centric summary detailing specific molecular effectors and supporting references, readers are referred to **Supplementary Table S1**.

**Table 1:** Conserved and species-specific immune evasion mechanisms in major invasive fungal pathogens.

Mechanism type	Exemplar pathogen	Key effector/strategy	Primary immune consequence	Potential therapeutic implications
Conserved Strategies	*C. albicans*	β-glucan masking via surface glycosylation	Impairs recognition by Dectin-1 and other PRRs	β-glucan unmasking agents (e.g., caspofungin); PRR agonists (e.g., Dectin−1 activators) to enhance immune recognition and synergize with checkpoint inhibitors or CAR−T cells (see Section 4.1).
	Multiple species (e.g., *C. albicans*, *A. fumigatus*)	Structured biofilm (ECM) formation	Creates physical barrier, limits immune cell infiltration & drug penetration	ECM−disrupting enzymes (e.g., glycoside hydrolases); enhanced drug delivery systems (e.g., nanoparticle−based antifungals); combinatorial use with cytokines (e.g., GM−CSF) to promote neutrophil infiltration (see Section 4.4).
	*A. fumigatus*	High-affinity iron acquisition (siderophores)	Depletes host iron, impairing iron-dependent antimicrobial effectors	Iron chelators (e.g., deferasirox); immunotherapy to bolster iron−withholding defenses (e.g., lipocalin−2−based strategies) (see Section 2.3).
Species-Specific Strategies	*C. neoformans*	GXM polysaccharide capsule; binding of complement regulator Factor H	Physically blocks phagocytosis; inhibits complement activation and opsonization	Anti−GXM monoclonal antibodies (e.g., 18B7); capsule biosynthesis inhibitors; complement−enhancing therapies (see Section 4.2).
	*A. fumigatus*	Secretion of gliotoxin	Inhibits macrophage phagocytosis & triggers apoptosis via PI3K/PIP3 disruption	Gliotoxin−neutralizing antibodies; PI3K pathway modulators; combinatorial regimens with PD−1 blockade (see Section 4.1).
	*C. albicans*	Secretion of acetate during hyphal growth	Reprograms macrophages towards an anti-inflammatory (M2) phenotype	Metabolic inhibitors (e.g., acetate−pathway blockers); M1−polarizing cytokines (e.g., IFN−γ, GM−CSF) to restore macrophage fungicidal activity (see Section 2.3).
	Members of the Mucorales order	Chitin/chitosan-rich cell wall	Confers resistance to host enzymatic degradation; alters immune recognition	Cell wall synthase inhibitors; enhanced innate sensing agonists (e.g., chitin−binding lectin−based activators); adjunctive use with antifungal−immunotherapy combinations (see Section 2.1).

Conserved strategies are employed by multiple fungal pathogens and represent broad targets for immunomodulation. Species−specific strategies are unique adaptations of particular pathogens, necessitating targeted therapeutic approaches.

ECM, extracellular matrix; GXM, glucuronoxylomannan; PRR, pattern recognition receptor.

For an extended pathogen−centric summary, see **Supplementary Table S1**.

## 3 Consequences of evasion: immune dysregulation in the immunocompromised host

Following the detailed examination of fungal immune evasion tactics (Section 2), this section focuses on their clinical sequelae: the profound and often paradoxical immune dysregulation that characterizes the immunocompromised host. As summarized in **Table 1**, these evasion strategies not only permit microbial persistence but also actively sculpt a dysfunctional immune microenvironment. In this context, host defense is characterized not by a simple functional deficit, but by a paradoxical imbalance that simultaneously cripples pathogen clearance and drives immunopathology—a vicious cycle central to the high morbidity and mortality of IFIs.

### 3.1 Dysregulation of innate immunity: from hypoactivity to hyperinflammation

The innate immune response in immunocompromised hosts is marked by a paradoxical imbalance between functional suppression and excessive, non-resolving inflammation. This duality is highly context-dependent, shaped by the specific nature of the host’s immunodeficiency, the infecting fungal pathogen, and the disease stage. A pivotal and frequently dysregulated pathway in this setting is the NOD-, LRR-, and pyrin domain-containing protein 3 (NLRP3) inflammasome. Although fungal components such as β-glucans and toxins like Candidalysin (Section 2.4) serve as physiological triggers for NLRP3 activation (14, 33, 38), the immunocompromised milieu often leads to a loss of regulatory control. This dysregulation results in excessive and sustained secretion of pro-inflammatory cytokines IL-1β and IL-18, fueling a damaging hyperinflammatory response that exacerbates tissue damage and correlates with poor clinical outcomes (39, 40). Concurrently, macrophage function is frequently skewed toward an anti-inflammatory, pro-repair (M2) phenotype in chronic or disseminated fungal infections (41). While this polarization may limit acute immunopathology, it impairs fungal killing capacity, thereby facilitating pathogen persistence and dissemination (42).

This dysregulated innate landscape manifests distinctly across different immunocompromised populations. In hematopoietic stem cell transplant (HSCT) recipients, delayed immune reconstitution and therapy-related tissue damage create a permissive niche where NLRP3-driven hyperinflammation poses a significant risk. Consequently, therapeutic immune augmentation in these patients must be carefully balanced against the potential for exacerbating graft-versus-host disease (GVHD) (43, 44). Conversely, in patients with autoimmune diseases on chronic immunosuppressants, the baseline inflammatory state is pharmacologically suppressed, often blunting the symptomatic presentation of fungal infections and delaying diagnosis—a critical consideration for clinical vigilance.

### 3.2 Deficiencies in adaptive immunity: impaired T helper and humoral responses

Adaptive immune defects are cornerstone vulnerabilities in immunocompromised hosts. Protective T-helper cell responses, particularly T helper type 1 (Th1) and type 17 (Th17), are frequently attenuated in conditions such as HIV/AIDS, hematological malignancies, and post-transplantation. This leads to impaired production of key antifungal cytokines such as interferon-gamma (IFN-γ) and interleukin-17 (IL-17), which are crucial for phagocyte activation and mucosal immunity (15, 16). This impairment is often compounded by an expansion of regulatory T cells (Tregs), which further suppress effector T cell function (45).

Humoral immunity is also compromised. Impaired generation and function of memory B cells lead to inadequate long-term protective antibody responses and reduced vaccine efficacy, leaving the host vulnerable to recurrent or persistent infection (46, 47).

These adaptive deficits translate into characteristic clinical challenges. In advanced HIV/AIDS, profound CD4^+^ T cell depletion cripples cell-mediated immunity against pathogens like Cryptococcus neoformans, compounded by chronic immune activation and B cell dysfunction that further impair fungal clearance. Immune potentiation strategies in this context must navigate the dual risks of inadequate response due to the depth of immunodeficiency and the potential for triggering immune reconstitution inflammatory syndrome (IRIS) upon initiation of antifungal therapy (45, 48). Similarly, in HSCT recipients, the convergence of Th1/Th17 deficiency and functional neutrophil impairment creates a permissive environment for invasive molds such as Aspergillus fumigatus (43, 44).

### 3.3 Aberrant immune checkpoint expression and T-cell exhaustion

Chronic antigen exposure and an immunosuppressive microenvironment during IFIs can induce a state of T-cell exhaustion, akin to that observed in chronic viral infections and cancer. A key hallmark is the upregulation of inhibitory immune checkpoint molecules such as programmed cell death protein 1 (PD-1). In murine models of invasive pulmonary aspergillosis, CD4^+^ T cells exhibit elevated PD-1 expression, and its ligand PD-L1 is upregulated on antigen-presenting cells, contributing to T cell dysfunction and impaired fungal clearance. Preclinical studies have demonstrated that blockade of the PD-1/PD-L1 pathway can restore immune function and improve outcomes in these models, highlighting its potential pathogenic role (43, 49). However, clinical translation remains nascent, with early-phase trials reporting variable efficacy and significant safety concerns in immunocompromised populations (43, 44).

The clinical implications of checkpoint dysregulation are particularly pronounced in specific host contexts. In HSCT recipients, while PD-1/PD-L1 blockade could theoretically reverse T-cell exhaustion and improve fungal clearance, its application is tempered by the well-documented risk of exacerbating GVHD. Similarly, in patients with underlying autoimmune conditions, checkpoint inhibitor therapy carries a documented risk of precipitating severe disease flares, as observed in oncology populations with autoimmune histories. These population-specific risks underscore the necessity for stringent patient stratification and rigorous immune monitoring when considering checkpoint modulation in immunocompromised hosts.

### 3.4 The dual role of trained immunity

Beyond classical innate and adaptive dysregulation, the emerging concept of trained immunity adds a layer of complexity. This de facto memory of innate immune cells involves epigenetic and metabolic reprogramming of monocytes, macrophages, and natural killer cells following exposure to pathogens or their components (e.g., β-glucans), leading to enhanced nonspecific responses upon rechallenge (50, 51).

While this phenomenon suggests a strategy to broadly reinforce innate defenses in immunocompromised hosts (e.g., using low-virulence Candida strains to induce protection (50, 52)), it poses a double-edged sword. Persistent fungal antigen exposure may drive maladaptive training, leading to excessive inflammation or innate immune cell exhaustion(53). The impact of trained immunity on antifungal vaccine efficacy in hosts with impaired adaptive immunity remains an important area for future investigation.

**Figure 2:**
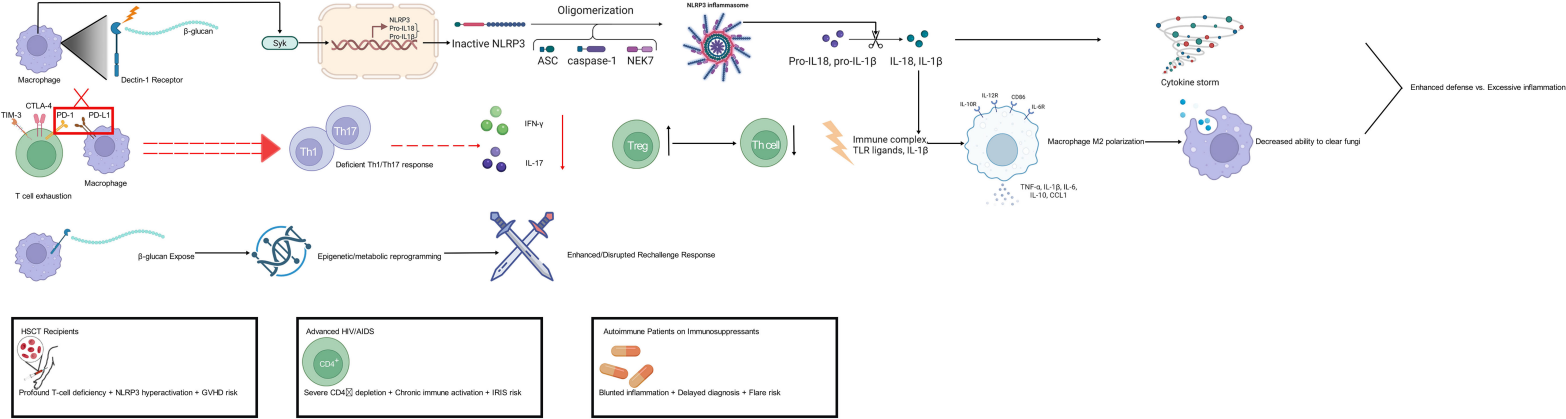
Dysregulated immune pathways in immunocompromised hosts during invasive fungal infections. Schematic representation of hyperinflammation (e.g., activation of the NLRP3 NOD−, LRR−, andpyrindomain−containing protein 3 inflammasome), adaptive immune deficiencies (impairment of T helper type 1 Th1 and type 17 Th17 responses), T-cell exhaustion (upregulation of programmed cell death protein 1 PD−1 and its ligand PD-L1), and trained immunity. These pathways illustrate the dual challenge of immune suppression and excessive inflammation, guiding patient-stratified immunomodulation.

Collectively, these interconnected pathways of innate and adaptive immune dysregulation—including aberrant inflammasome activation, impaired Th1/Th17 responses, T-cell exhaustion, and the dual role of trained immunity—create a complex, context-dependent landscape that both impairs fungal clearance and exacerbates tissue injury, providing both a rationale and a cautionary framework for immunomodulatory interventions. **Figure 2** provides a schematic overview of these dysregulated immune pathways in immunocompromised hosts during invasive fungal infections.

## 4 Harnessing host immunity: emerging immunotherapeutic strategies

The intricate interplay between fungal evasion mechanisms and host immune dysregulation, as detailed in Sections 2 and 3, not only elucidates the pathogenesis of invasive fungal infections (IFIs) but also reveals a spectrum of therapeutic vulnerabilities within the host immune system. This growing understanding has catalyzed a paradigm shift from purely pathogen-centric approaches toward strategies that aim to harness or augment host immunity. Building upon these mechanistic insights, a range of immunotherapeutic strategies has entered the translational pipeline, spanning from preclinical discovery to early-phase clinical investigation. These approaches are designed to correct specific immune deficits, enhance antifungal effector functions, and achieve targeted pathogen clearance, offering a promising complement to conventional antifungal regimens.

### 4.1 Immune potentiation and reconstitution therapies

Conserved fungal evasion strategies, particularly β-glucan masking and biofilm formation, present actionable targets for broad-spectrum immune enhancement (**Table 1**). For instance, pharmacological agents that unmask β-glucan, such as echinocandins (e.g., caspofungin), can synergize with immunotherapies by enhancing the visibility of the pathogen to innate immune cells, thereby promoting recognition and phagocytosis (7, 53).

#### 4.1.1 Cytokine therapy

The administration of recombinant cytokines aims to restore deficient immune effector pathways. Recombinant interferon-gamma (IFN-γ), a pivotal Th1 cytokine, enhances macrophage oxidative burst and phagocytic activity against a broad range of fungi, including Aspergillus and Candida species. It is approved as an adjunctive therapy for chronic granulomatous disease and has demonstrated clinical benefit in refractory IFIs (54). Similarly, granulocyte-macrophage colony-stimulating factor (GM-CSF) promotes the differentiation, survival, and antifungal activity of neutrophils and macrophages. Beyond its role in stimulating hematopoiesis, preclinical models have shown that GM-CSF can program hematopoietic stem and progenitor cells during *C. albicans* vaccination, conferring enhanced protection upon rechallenge (55).

The adjunctive use of cytokines such as IFN-γ and GM-CSF alongside conventional antifungals represents a potent synergistic strategy. IFN-γ primes macrophages and neutrophils, enhancing their oxidative burst and phagocytic capacity against fungi, thereby reducing fungal burden and limiting tissue invasion, which creates a more permissive environment for drug efficacy (54). In clinical practice, recombinant IFN-γ has been shown to improve outcomes in patients with acute cryptococcal meningitis when combined with amphotericin B-based therapy, showing a trend toward enhanced mycologic clearance (56).

However, the safety profile of cytokine therapy in immunocompromised hosts requires careful consideration. In a long-term follow-up study of patients with chronic granulomatous disease receiving IFN-γ prophylaxis, fever was the most common adverse event (occurring in 37% of patients), but no life-threatening IFN-γ-related adverse events or discernible effects on growth were observed (57). A recent meta-analysis confirmed the efficacy of IFN-γ in reducing serious infections in chronic granulomatous disease, with a risk ratio of 0.56 (95% CI 0.35-0.90), while noting that serious adverse events were rare (58). In the hematopoietic stem cell transplant (HSCT) setting, a retrospective study of 32 patients receiving adjuvant IFN-γ for IFIs reported that fever was common (28%), but there were no episodes of cytopenias or liver dysfunction attributable to IFN-γ therapy (59). Importantly, IFN-γ did not precipitate or exacerbate graft-versus-host disease (GVHD); in fact, some patients showed improvement in GVHD during therapy (59). These findings suggest that IFN-γ can be administered safely at doses of 50 μg subcutaneously three times weekly, though close monitoring for fever and constitutional symptoms is warranted (57, 59). Dose adjustments may be considered in patients experiencing significant adverse effects, though specific guidelines for combination with antifungal agents remain to be established in prospective trials.

#### 4.1.2 Immune checkpoint inhibitors

Preclinical models demonstrate that blockade of the PD-1/PD-L1 axis can restore exhausted T-cell function and, when combined with antifungals like caspofungin, significantly improve fungal clearance and survival in invasive pulmonary aspergillosis (49, 53). This multi-layered synergy operates through complementary mechanisms elucidated at the molecular level. The ICI reverses T-cell exhaustion by blocking the PD-1/PD-L1 axis, thereby restoring effector T-cell proliferation and cytokine production (e.g., IFN-γ) (43). Concurrently, caspofungin exerts immunomodulatory effects beyond its direct fungicidal activity. Subinhibitory concentrations of caspofungin have been shown to unmask immunostimulatory β-(1,3)-glucan in the *C. albicans* cell wall, a process driven by increased chitin synthesis in response to drug-induced stress (60). This unmasking occurs preferentially in filamentous cells and is regulated by the calcineurin and Mkc1 mitogen-activated protein kinase pathways, which work synergistically to remodel the cell wall architecture (60, 61). The exposed β-glucan becomes accessible to the pattern recognition receptor Dectin-1 on innate immune cells, enhancing phagocytosis and pro-inflammatory cytokine production (62). Thus, caspofungin treatment not only reduces fungal burden but also actively renders fungi more visible to the reinvigorated immune effectors, creating a coordinated antifungal response (53). This mechanistic understanding informs the optimal sequencing of combination therapy, as β-glucan exposure occurs rapidly following caspofungin exposure, potentially priming the immune system for subsequent ICI-mediated T-cell reinvigoration. This coordinated reinvigoration of adaptive and innate responses underlies the improved outcomes observed in preclinical models.

### 4.2 Vaccines and antibody-based therapies

These strategies aim to induce or provide pathogen-specific, adaptive immunity.

Vaccines represent a critical strategy to induce durable, protective immune memory against invasive fungal infections. Promising candidates are in development across diverse platforms. For instance, the recombinant subunit vaccine NDV-3A demonstrated efficacy in a Phase II trial for recurrent vulvovaginal candidiasis (63), while whole-cell attenuated vaccines, such as the heat-killed *C. neoformans* Δsgl1 mutant (which lacks sterylglucosides), have shown robust protection in immunocompromised murine models (64, 65). Similarly, vaccines based on chitosan-deficient *C. neoformans* strains have also demonstrated efficacy (65). Moreover, NDV-3A also protects against multidrug-resistant *Candida auris* in murine models, highlighting its broad-spectrum potential (66). In cryptococcosis, several innovative approaches have shown robust protection in preclinical models, including vaccines that utilize glucan particles for targeted delivery of recombinant antigens, as well as whole−cell vaccines based on heat−killed, attenuated strains. For aspergillosis, the *A. fumigatus* ΔsglA mutant has shown promise as a live attenuated vaccine (67). These platforms have elicited protective responses even in immunocompromised murine models, underscoring their potential for high−risk hosts (65, 67). A comprehensive comparison of vaccine candidates—spanning subunit, recombinant, whole−cell, and vectored platforms—is provided in **Supplementary Table S2**, which details their target antigens, developmental stages, and evidence across major fungal pathogens. Subunit vaccines like NDV−3A offer high specificity and safety for clinical advancement, while whole−cell attenuated vaccines may provide broader antigenic exposure and have shown compelling efficacy in preclinical studies of aspergillosis and cryptococcosis (63, 65, 67). This comparative landscape underscores the diversity of vaccine strategies and highlights candidates with potential for translation into immunocompromised populations.

Monoclonal Antibodies (mAbs): These agents confer immediate, passive immunity through the neutralization of virulence factors or opsonization of pathogens. Early efforts like the anti-Hsp90 monoclonal antibody faced developmental challenges, while recent approaches focus on virulence factor neutralization and opsonization (20, 68, 69). Current focus includes antibodies against polysaccharide capsules (e.g., anti-GXM mAbs for Cryptococcus), which can promote phagocytosis and are protective in animal models (20, 70). Similarly, mAbs targeting cell wall proteins like Als3 or Hyr1 of *C. albicans* have shown protective efficacy in preclinical studies (68, 69). The therapeutic potential of bispecific antibodies or antibody-drug conjugates in mycology remains largely unexplored.

**Supplementary Table S2** summarizes key vaccine candidates, including NDV-3A for candidiasis (63), *A. fumigatus* ΔsglA mutant for aspergillosis (60), and attenuated or antigen-deficient strains, such as the *C. neoformans* Δsgl1 mutant (66) or chitosan-deficient strains (65). Notably, while subunit vaccines such as NDV-3A show promise in clinical trials for candidiasis, whole-cell or attenuated vaccines (e.g., Δsgl1 strain for aspergillosis) have demonstrated robust protection in preclinical models, particularly in immunocompromised hosts. This diversity in vaccine platforms underscores the need for pathogen- and population-specific design.

Beyond single-agent use, the synergistic potential of vaccines with immune checkpoint inhibitors is emerging as a promising strategy to enhance adaptive immunity. Drawing from oncology, where a phase 1/2 trial of an IDO/PD−L1−targeting immunomodulatory vaccine combined with anti−PD−1 (nivolumab) demonstrated enhanced antitumor responses in metastatic melanoma (71), this paradigm supports exploration in mycology. Coupling fungal antigen−specific vaccines (e.g., NDV−3A or Δsgl1−based vaccines) with PD−1/PD−L1 blockade could simultaneously boost pathogen−specific T−cell priming and reverse exhaustion, potentially achieving more durable protection in immunocompromised hosts. Clinical translation of this synergy, however, is complicated by unique safety concerns discussed in Section 7.1.

### 4.3 Adoptive cellular therapies

This approach involves the ex vivo expansion and reinfusion of immune cells with antifungal activity.

Fungus-Specific T Cells: Adoptive transfer of T cells expanded against fungal antigens (e.g., *Aspergillus*- or *Candida*-derived peptides) can reconstitute pathogen-specific cellular immunity. Clinical-scale manufacturing of these cells is feasible, and early-phase studies in hematopoietic stem cell transplant recipients have suggested safety and potential efficacy (72, 73).

Chimeric Antigen Receptor (CAR) T Cells: Engineering T cells to express CARs targeting fungal surface antigens represents a cutting-edge strategy. Preclinical proof-of-concept has been achieved for *A. fumigatus* and *C. neoformans*. CAR-T cells targeting *Aspergillus* surface epitopes effectively reduced fungal burden and improved survival in mouse models of invasive aspergillosis (74, 75). Similarly, CAR-T cells engineered to recognize cryptococcal GXM capsule components exhibited antifungal activity *in vitro* and *in vivo* (70, 76). While safety concerns (e.g., cytokine release syndrome, on-target/off-tumor effects) and logistical hurdles are significant, CAR-T therapy offers the potential for highly specific and potent antifungal activity.

The efficacy of adoptive cellular therapies may be further enhanced through combination with strategies that disrupt fungal protective niches. For instance, immunotherapies that enhance neutrophil recruitment or antibodies targeting biofilm matrix components (e.g., β-glucan) could disrupt biofilm architecture, increasing fungal susceptibility to CAR-T cells and re-exposing concealed PAMPs to immune surveillance (23, 24). Similarly, combining iron chelators to starve the pathogen with adoptive T-cell therapy could simultaneously address a key virulence mechanism of *A. fumigatus* while bolstering immune effector function (27).

**Figure 3:**
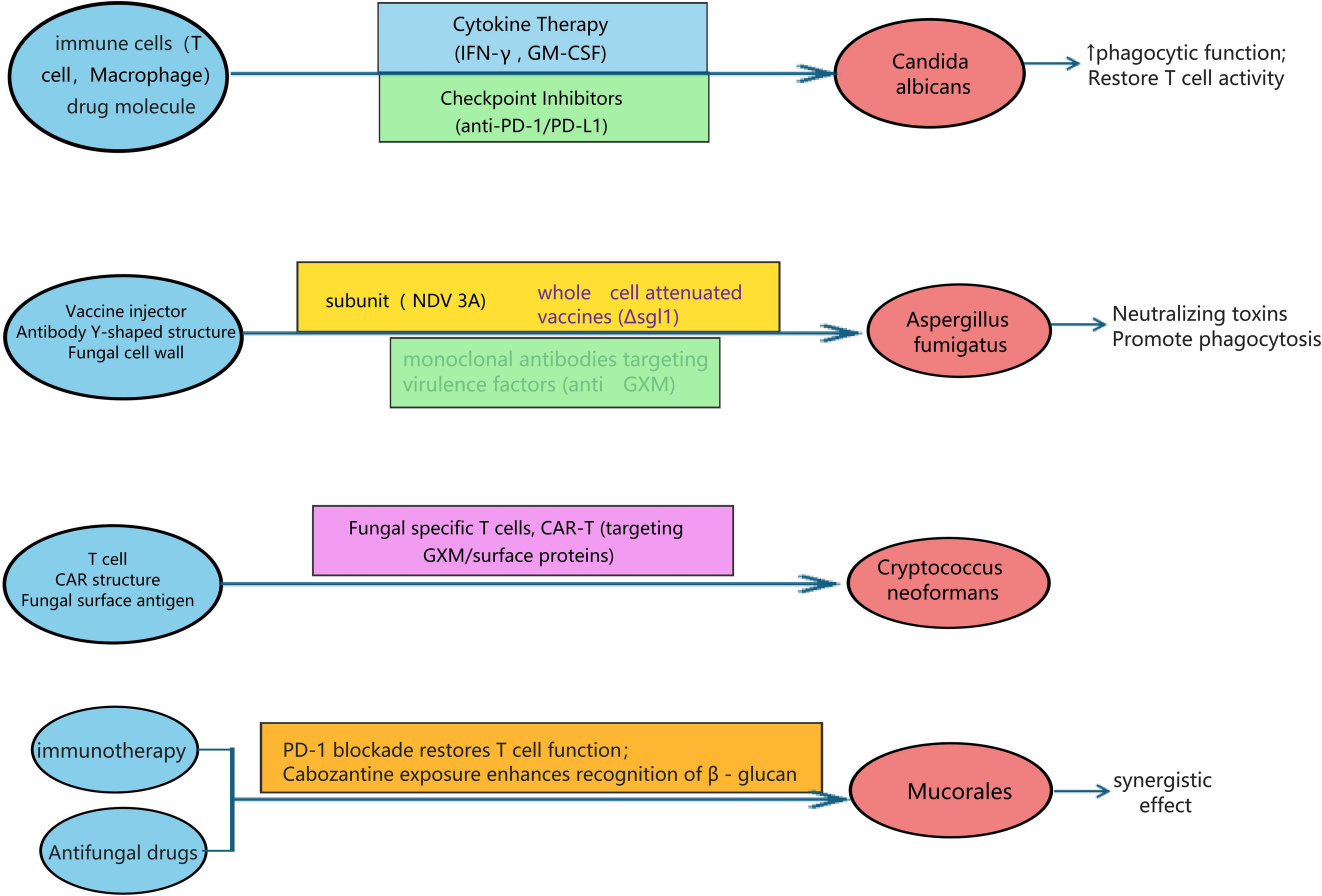
Emerging immunotherapeutic strategies against invasive fungal infections. Overview of cytokine therapy, immune checkpoint inhibitors, monoclonal antibodies, vaccines, and adoptive cellular therapies (including CAR-T cells). The integration of these modalities with conventional antifungals represents a paradigm shift toward host-pathogen integrated management.

**Figure 3** provides a schematic overview of these major immunotherapeutic strategies, illustrating their cellular and molecular targets. While the current evidence—primarily from preclinical and early-phase clinical studies—strongly supports the rationale for integrating these modalities into combinatorial regimens alongside traditional antifungals, the optimal application of these strategies necessitates a precision medicine approach. This requires careful consideration of the host’s specific immune defect, the fungal pathogen involved, and the potential risks of immunopathology—challenges addressed in the following section.

## 5 Translational challenges and the path to precision immunotherapy

### 5.1 Limitations of current evidence and heightened risks in immunocompromised hosts

Despite the compelling preclinical rationale for various immunotherapies outlined in Section 5, their translation into safe and effective clinical practice for immunocompromised hosts faces distinct and formidable hurdles. The translational validity of current mechanistic insights is constrained by model systems. First, many foundational studies utilize laboratory-adapted fungal strains (e.g., *C. albicans SC5314*), which may not fully reflect the genetic diversity, phenotypic plasticity, or virulence repertoire of contemporary clinical isolates (17, 18). Emerging evidence reveals substantial heterogeneity among clinical isolates that challenges the generalizability of findings derived from single reference strains.

For *C. albicans*, laboratory strains differ significantly from clinical isolates in host immune interactions. Gerwien et al. compared vaginal isolates from symptomatic and asymptomatic women with the laboratory strain *SC5314* (77). They found marked differences during macrophage interaction. Although clinical strains responded similarly to *SC5314* in standard assays, they showed reduced β-glucan exposure on the cell surface. This property was partially lost under vaginal niche-like nutrient conditions (77). Macrophage damage, phagocytosis survival, and filamentation capacities were also highly strain-specific (77). These findings highlight critical heterogeneity that must be considered when translating laboratory findings to patient outcomes.

For *A. fumigatus*, strain heterogeneity directly influences virulence outcomes. Kowalski, Beattie et al. showed that the two most common reference strains, *AF293* and *CEA10*, differ significantly in infection-related traits (78). CEA10 exhibited greater fitness under hypoxic growth and higher lethality in murine models due to enhanced tissue invasion (78). When the authors analyzed environmental and clinical isolates, they observed wide variation in hypoxic fitness and mouse lethality. This variation mirrored the *AF293-CEA10* dichotomy (78). Strikingly, experimental evolution of *AF293* under hypoxia for 20 generations increased its fitness and virulence (78). This demonstrates that laboratory passage can select for phenotypic changes that do not reflect clinical reality. Dos Santos et al. further characterized 15 clinical strains of *A. fumigatus* and cryptic species, finding heterogeneity in drug susceptibility and virulence within each species, with known virulence-related genes showing variability in paralog number across strains (79).

For *C. parapsilosis*, strain-specific virulence factor expression has been documented. Gandra et al. examined secreted aspartic protease (Sapp1 and Sapp2) production in 10 clinical isolates (80). Protease activity ranged from 44.15 to 270.61 fluorescence units, with Sapp1 surface levels up to fourfold higher than Sapp2 (80). This heterogeneity in a key virulence factor has direct implications for drug development, as Saps are potential therapeutic targets (80).

These findings underscore a critical translational challenge: discoveries made using single reference strains must be validated across panels of diverse clinical isolates due to their inherent genetic, phenotypic, and regulatory variability. Moreover, the host-dependent nature of virulence observed in *A. fumigatus* studies (80) highlights the need for multiple infection models that recapitulate different aspects of human disease. Future research should prioritize comparative genomic and phenotypic analyses of large clinical isolate collections to identify conserved pathogenic mechanisms that are truly generalizable and therefore suitable as therapeutic targets.

Second, a significant portion of efficacy data, particularly for strategies like checkpoint inhibitors, derives from immunocompetent murine models of acute infection (43, 49). While invaluable for establishing proof-of-concept, these models often fail to recapitulate the nuanced, chronic, and iatrogenic immune dysfunction—such as prolonged lymphopenia or complex immunosuppressive regimens—that defines human at-risk cohorts (44). This gap limits the predictive validity of such models for clinical outcomes in profoundly immunocompromised patients.

Real-world clinical data have begun to elucidate the true magnitude of these risks. In patients receiving chimeric antigen receptor (CAR) T-cell therapy, invasive fungal diseases (IFDs) have been documented as a significant post-treatment complication, with global incidence rates ranging from 0% to 10% (81). A recent systematic review of post-CAR-T fungal infections reported that *Aspergillus* spp.*, Candida* spp., and *Pneumocystis jirovecii* are the predominant pathogens, with most infections occurring within the first 60 days following T-cell infusion, although cases occurring up to one year post-treatment have been described (81). Multiple risk factors contribute to this vulnerability, including underlying hematological malignancies, lymphodepleting chemotherapy, pre-existing leukopenia and hypogammaglobulinemia, and the use of high-dose corticosteroids and interleukin-6 blockers (e.g., tocilizumab) for management of cytokine release syndrome (CRS) and immune effector cell-associated neurotoxicity syndrome (ICANS) (81).

Case reports further underscore the clinical significance of this risk. Hou et al. described a 17-year-old male with acute lymphoblastic leukemia who developed a fatal invasive fungal infection following CAR-T cell therapy, highlighting the need for vigilance particularly in patients with pre-existing fungal infections and those receiving immunosuppressive agents for CRS management (82). This case emphasizes that early surgical intervention may be more effective than antifungal monotherapy in some patients with invasive fungal infections (82).

For immune checkpoint inhibitors, fungal infections appear to be less common. Bernardes et al. reviewed the literature and found that most reported cases involve invasive aspergillosis and Pneumocystis pneumonia, typically occurring in patients requiring high-dose corticosteroids for the management of immune-related adverse events (irAEs) (83). Importantly, treatment-related toxicities that necessitate prolonged immunosuppressive therapy appear to play a key role in the development of fungal infections during immunotherapy, highlighting the interconnected nature of treatment efficacy, toxicity management, and infectious risk (83).

Several pragmatic questions lack evidence-based answers. The optimal timing and sequencing of immunomodulators relative to antifungal agents and baseline immunosuppressants remain empirical (44). Furthermore, strategies reliant on adaptive immune memory, such as vaccines or certain monoclonal antibodies, may have inherently limited efficacy in hosts with profound B- and T-cell deficiencies (46). Pathogen evolution under immune pressure, including antigenic variation or capsule remodeling, also poses a threat to the durability of such interventions (84).

### 5.2 Bridging the gap: key challenges in clinical translation

Beyond the limitations of preclinical models, the foremost hurdle in clinical application is the amplified risk of iatrogenic immunopathology in hosts with pre-existing immune dysregulation. For instance, while PD-1 blockade synergized with caspofungin in a murine model of invasive pulmonary aspergillosis (49), its application in hematopoietic stem cell transplant (HSCT) recipients is tempered by the grave risk of exacerbating graft-versus-host disease (GVHD) (44).

Immune Checkpoint Inhibitors: A Paradigm of Translational Dilemma.

Managing cytokine release syndrome (CRS) presents a major clinical challenge with direct implications for fungal infection risk. CRS is a hyperinflammatory state triggered by CAR-T cell therapy and bispecific T-cell engagers. It is characterized by excessive cytokine release that often mimics infectious conditions, complicating diagnosis and treatment (85). A systematic review by Arvanitis et al. found that infections occur in up to 23% of patients post-CAR-T therapy and 24% post-BiTE therapy, with fungal pathogens including *Candida* spp.*, Aspergillus* spp., and *Pneumocystis jirovecii* among the documented causes (85). Critically, the immunosuppressive therapies used to manage CRS—including tocilizumab and corticosteroids—further elevate the risk of secondary infections, creating a therapeutic paradox where treatment of immunotherapy toxicity increases susceptibility to opportunistic pathogens (85).

The temporal relationship between CRS management and infectious complications is particularly noteworthy. Patients requiring high-dose corticosteroids for severe CRS or ICANS experience prolonged immunosuppression that extends the window of vulnerability to fungal infections (81). This underscores the need for a multidisciplinary approach involving close collaboration between oncology and infectious disease specialists, with dedicated infectious disease input and pre-treatment evaluation being essential for optimizing patient outcomes (85).

In the context of allogeneic hematopoietic stem cell transplantation, additional considerations regarding drug-drug interactions emerge. Farina et al. demonstrated in a cohort of 51 HSCT recipients that coadministration of the broad-spectrum triazole isavuconazole with the mTOR inhibitor sirolimus was safe and feasible, with no toxicities related to drug-drug interaction observed in patients achieving concomitant therapeutic levels of both drugs (86). This real-world evidence supports the feasibility of combining antifungal prophylaxis with immunosuppressive regimens, though therapeutic drug monitoring is essential to ensure safety (86).

In hematopoietic stem cell transplant (HSCT) recipients—a population at high risk for invasive mold infections—PD−1/PD−L1 inhibition carries a well−documented danger of exacerbating graft−versus−host disease (GVHD) (44). Recent preclinical studies in invasive pulmonary mucormycosis have begun to address the question of timing, demonstrating that early adjunct anti-PD-L1 therapy (initiated concurrently with antifungal therapy) promoted significantly stronger innate immune cell activation, reinvigoration of T-helper-cell signaling, and reversal of infection-induced exhaustion signals compared to delayed ICI administration (87). These findings suggest that the window for optimal ICI intervention may be early in the course of infection, before the establishment of sustained immune paralysis. However, this must be balanced against the risk of exacerbating GVHD in the post-transplant setting, highlighting the need for carefully designed clinical trials with serial immune monitoring to define the optimal therapeutic window. Similarly, in patients with advanced HIV/AIDS or autoimmune conditions, therapeutic immune potentiation may trigger immune reconstitution inflammatory syndrome (IRIS) or flare underlying autoimmunity (44, 48). These risks are poorly captured in standard immunocompetent mouse models, highlighting a critical disconnect between preclinical efficacy and clinical safety. Therefore, while ICIs represent a rationally compelling strategy, their use must be guided by stringent patient stratification, rigorous immune monitoring, and protocols designed to mitigate unique iatrogenic immunopathology (44).

### 5.3 Tools for precision: multi-omics and dynamic immune profiling

Overcoming these challenges necessitates a move beyond a “one-size-fits-all” approach. Precision immunotherapy requires deep, multidimensional characterization of the host-pathogen interface, enabled by cutting-edge technologies.

High-Resolution Immune Mapping: Single-cell RNA sequencing (scRNA-seq) and spatial transcriptomics are poised to unravel the spatiotemporal dynamics of immune-fungal interactions at unprecedented resolution. For example, spatial transcriptomic approaches have begun mapping immune cell niches and pathogen localization in other infectious and inflammatory contexts, providing a direct framework for application in fungal diseases (53). In mycology, scRNA-seq of infected tissues can identify functionally distinct immune subsets, such as immunosuppressive monocyte populations, and correlate their state with checkpoint ligand expression (49). Integrating these modalities will pinpoint immune-evasion niches within lesions, directly informing the rationale for targeted immunotherapeutic interventions. For instance, spatial profiling of cryptococcal granulomas could reveal localized zones of immune checkpoint upregulation, guiding targeted immunotherapies. These insights identify novel, spatially resolved cellular targets for intervention. Spatial transcriptomics, though underexplored in mycology, holds immense potential for mapping immune evasion niches and host-pathogen interactions within infected tissues (53). This approach has begun to reveal spatially resolved immune responses in other infectious and inflammatory contexts, providing a framework for its application in fungal diseases. Integrating these high-resolution datasets with clinical metadata through advanced computational pipelines and machine learning models is essential to build predictive algorithms (88, 89). These tools can define the ‘right drug, right patient, right time’ paradigm by matching individual patients with optimal combinatorial regimens, and must ultimately transition from research applications to components of routine clinical management.

Dynamic Host Immune Phenotyping: Clinical immune monitoring must evolve from static assessments to dynamic profiling. Integrating parameters such as T-cell exhaustion markers (PD-1, TIM-3), inflammasome activation status (IL-1β, IL-18), functional assays of phagocyte activity, and pathogen-specific T-cell reconstitution can stratify patients into those needing immune enhancement versus those requiring anti-inflammatory control (40, 43). Furthermore, host genetic polymorphisms in immune genes, such as caspase recruitment domain family member 9 (CARD9), the β-glucan receptor Dectin-1 (encoded by CLEC7A), and signal transducer and activator of transcription 3 (STAT3) significantly influence susceptibility and therapeutic response (90, 91), advocating for pre-emptive genotyping to guide prophylactic or therapeutic choices.

Pathogen-Specific Characterization: Therapeutic decisions must be informed by the fungal species, strain, antifungal resistance profile, and expression of key virulence factors. Genomic and transcriptomic analysis of clinical isolates can identify strain-specific adaptations that may influence immunotherapy efficacy (84, 92).

### 5.4 Toward personalized regimens: integrating host, pathogen, and clinical context

The ultimate goal is to integrate these multidimensional datasets to guide personalized therapy. This involves:

1. Computational Integration and Predictive Modeling: Advanced computational pipelines and machine learning models are required to fuse longitudinal multi-omics data (host transcriptomics, proteomics, pathogen genomics) with clinical metadata (83, 85). This integration can build predictive algorithms to match individual patients with optimal combinatorial regimens, defining the “right drug, right patient, right time” paradigm.
2. Biomarker-Driven Clinical Trial Design: Future clinical trials must adopt adaptive designs incorporating rigorous, serial immune monitoring as integral endpoints. Trials evaluating ICIs for invasive aspergillosis in HSCT recipients, for example, should stratify patients based on dynamic biomarkers including T-cell exhaustion markers (PD-1, TIM-3), pathogen-specific T-cell reconstitution, inflammasome activation (e.g., interleukin [IL]-1β and IL-18), and host genetic polymorphisms (e.g., in CARD9, Dectin-1) (43, 44, 90). The role of additional inhibitory receptors, such as TIM-3, in fungal infection-associated T cell exhaustion warrants further investigation, as they may serve as predictive biomarkers alongside PD-1 (92). This precision-guided enrollment will help identify patients most likely to benefit while minimizing risks like GVHD exacerbation or IRIS (44, 48).
3. Engineering Next-Generation Therapeutics: To address the safety concerns outlined above (e.g., CRS, on-target/off-tumor effects), therapeutic platforms must evolve toward greater controllability. This includes developing CAR-T cells with ‘suicide switches’ or logic-gated activation systems to mitigate risks such as cytokine release syndrome (75), exploring mRNA-based vaccine platforms to overcome adaptive immune hyporesponsiveness in severely lymphopenic hosts (93), and engineering bispecific antibodies or antibody-drug conjugates targeting fungal virulence factors (e.g., GXM, gliotoxin) for enhanced specificity and potency (20, 69).
4. Fostering Interdisciplinary Convergence: Overcoming the translational challenges in antifungal immunotherapy requires concerted collaboration across immunology, microbiology, clinical mycology, data science, and bioengineering. Establishing standardized biorepositories of clinical fungal isolates, developing reproducible immune monitoring panels, and conducting well-designed adaptive clinical trials with integrated translational endpoints will be essential to accelerate progress in this field (44).
5. Structured Safety Monitoring Protocols: Given the significant infectious risks of immunomodulatory therapies, structured safety monitoring is essential. Key components include:
  - Pre-treatment screening for latent fungal infections, especially in patients with prior fungal disease or those from endemic areas (82).
  - Close monitoring for CRS and ICANS during therapy, with prompt diagnostic workup to distinguish inflammatory syndromes from active infection (85).
  - Risk-adapted antifungal prophylaxis, including mold-active prophylaxis for high-risk CAR-T cell recipients or those requiring prolonged high-dose corticosteroids for irAE management (81, 83).
  - Therapeutic drug monitoring when combining antifungal agents with immunosuppressive drugs that have narrow therapeutic windows or known interactions, such as isavuconazole with sirolimus (86).

These safety considerations, which vary considerably across immunotherapeutic modalities, are summarized in **Table 2**, providing a practical framework for risk stratification and clinical management.

**Table 2:** Clinical safety considerations for immunotherapy in immunocompromised hosts.

Immunotherapy modality	Reported IFD incidence	Key pathogens	Primary risk factors	Management strategies	References
CAR-T Cell Therapy	0-10% globally	*Aspergillus* spp., *Candida* spp., *Pneumocystis jirovecii*	Underlying malignancy, lymphodepleting chemotherapy, CRS/ICANS management (corticosteroids, tocilizumab)	Pre-treatment screening, risk-adapted prophylaxis, early surgical intervention for localized IFD	(81, 82)
Immune Checkpoint Inhibitors	Uncommon	Invasive aspergillosis, Pneumocystis pneumonia	High-dose corticosteroids for irAE management	Cautious corticosteroid use, rapid tapering when possible, consider prophylaxis during prolonged immunosuppression	(83)
Cytokine Therapy (IFN-γ)	N/A (used as treatment, not associated with increased IFD risk)	N/A	Fever (common, 28-37%), rare serious adverse events	Dose 50 μg SC three times weekly, monitor for fever and constitutional symptoms	(94, 95)
Combination Therapy (Isavuconazole + Sirolimus)	N/A (antifungal + immunosuppressant)	N/A	Potential drug-drug interactions	Therapeutic drug monitoring essential, no significant toxicity observed with concomitant therapeutic levels	(86)

In conclusion, the path to successful immunotherapy for IFIs in immunocompromised hosts lies in embracing precision medicine. By systematically addressing translational challenges through biomarker-driven research and leveraging multi-omics tools to deconvolute the complex host-pathogen-immune triad, the field can transform broad immunomodulatory concepts into safe, effective, and personalized treatments that significantly improve patient outcomes.

## 6 Conclusion

The clinical management of invasive fungal infections (IFIs) in immunocompromised hosts is undergoing a fundamental transformation, driven by an increasingly sophisticated understanding of the dynamic interplay between fungal immune evasion and host immune dysregulation. This review has synthesized evidence from the past decade, underscoring that effective long-term control of IFIs necessitates a paradigm shift from a purely pathogen-centric approach to an integrated “direct antifungal + host-directed immunomodulation” dual-track strategy.

Invasive fungal infections represent a quintessential example of how pathogen success is defined not only by intrinsic virulence factors but also by the ability to exploit and distort host immunity. The conserved and species-specific immune evasion strategies employed by fungi such as *C. albicans*, *A. fumigatus*, and *C. neoformans* directly contribute to the profound immune dysregulation—characterized by deleterious hyperinflammation, deficient Th1/Th17 responses, T-cell exhaustion, and checkpoint upregulation—that defines the immunocompromised host. This intertwined pathophysiology fundamentally limits the efficacy of conventional antifungal monotherapy.

Consequently, the future of antifungal therapy lies in a synergistic, dual-target approach: simultaneously reducing the fungal burden with direct antifungal agents and rectifying the underlying host immune defect with tailored immunomodulation. The array of emerging strategies, from broad immune potentiators like IFN-γ and checkpoint inhibitors to highly specific agents such as pathogen-directed monoclonal antibodies and CAR-T cells, provides a versatile therapeutic toolkit (43, 54, 68, 75). However, their successful application must be guided by the principles of precision medicine. Clinical success will depend on our ability to dynamically decode the specific “immunodeficiency signature” of the individual host, the antigenic and metabolic profile of the infecting pathogen, and the complex interplay between them.

By systematically addressing translational challenges through biomarker-driven research and leveraging cutting-edge multi-omics tools, the field can transform broad immunomodulatory concepts into safe, effective, and personalized treatments. This evolution from a pathogen-centric to a host-pathogen integrated therapeutic framework holds the promise of significantly improving the dire prognosis faced by immunocompromised patients with invasive fungal infections.
