# Supplementary material for: Immune evasion, dysregulation, and emerging immunotherapies for invasive fungal infections in the immunocompromised host
Source: Front Immunol. 2026 Apr 1;17:1788786. doi: 10.3389/fimmu.2026.1788786 (PMC13079191; doi:10.3389/fimmu.2026.1788786)
Supplement: Supplementary file 1 [file Table1.docx]

Supplementary Table S1. Comprehensive summary of immune evasion mechanisms and key virulence factors in invasive fungal pathogens.

| Pathogen | Evasion Mechanism Category | Key Molecular Effector / Strategy | Functional Role in Evasion | Selected References |
| --- | --- | --- | --- | --- |
| Candida albicans | Surface Antigen Masking | O‑/N‑glycosylation of cell wall mannoproteins | Masks underlying β‑(1,3)‑glucan from Dectin‑1 recognition; clinical isolates show enhanced antigenic variation | [7, 9, 17, 18] |
|  | Biofilm Formation | Adhesins (Als3, Hwp1); ECM (β‑glucans, eDNA) | Stabilizes biofilm architecture, impedes immune‑cell infiltration, enhances antifungal resistance | [23, 24, 26] |
|  | Metabolic Interference | Secretion of metabolites (e.g., acetate) during adaptation. | Reprograms host metabolism and immune responses to facilitate persistence and disease tolerance. | [30] |
|  | Effector Secretion | Secreted aspartyl proteases (Saps); Candidalysin (from Ece1) | Degrades complement components (e.g., C3b, C5a); induces epithelial damage and dysregulated NLRP3 inflammasome activation | [31-34] |
| Aspergillus fumigatus | Surface Modulation | Conidial surface protein CcpA | Masks immunogenic epitopes; essential for virulence and early immune evasion | [19] |
|  | Biofilm Formation | ECM rich in galactomannan and hydrophobic proteins | Confers resistance in lung cavities or on biotic/abiotic surfaces; limits drug penetration and immune‑cell access | [24, 25] |
|  | Nutrient Competition | High‑affinity siderophores (e.g., ferricrocin, triacetylfusarinine C) | Scavenges host iron, impairing iron‑dependent antimicrobial effectors | [27, 28] |
|  | Effector Secretion | Gliotoxin | Disrupts macrophage PI3K/PIP3 homeostasis, inhibits phagosome maturation, and induces apoptosis | [12,13] |
| Cryptococcus neoformans | Physical Barrier & Molecular Mimicry | GXM polysaccharide capsule; binding of host Factor H | Physically blocks phagocytosis; inhibits complement activation and opsonization via co‑option of host regulatory proteins | [11, 20, 21] |
| Mucorales spp. | Structural Resistance | Chitin‑ and chitosan‑rich cell wall | Confers resistance to host enzymatic degradation; alters immune recognition patterns | [22] |

Notes:

- This table provides an extended, pathogen‑centric listing of immune evasion mechanisms discussed in Sections 2.1–2.4. It complements the synthesized, mechanism‑type overview in Table 1.

- ECM: extracellular matrix; eDNA: extracellular DNA; GXM: glucuronoxylomannan.

- Species names are italicized in accordance with microbiological nomenclature.

- References include key studies from 2015–2025, with emphasis on recent advances in immune‑evasion mechanisms and therapeutic targeting.
