# Supplementary material for: Immune evasion, dysregulation, and emerging immunotherapies for invasive fungal infections in the immunocompromised host
Source: Front Immunol. 2026 Apr 1;17:1788786. doi: 10.3389/fimmu.2026.1788786 (PMC13079191; doi:10.3389/fimmu.2026.1788786)
Supplement: Supplementary file 2 [file Table2.docx]

Supplementary Table S2. Promising vaccine candidates against invasive fungal infections: platforms, antigens, and development status.

| Target Pathogen | Vaccine Candidate / Type | Target Antigen(s) | Platform Description | Development Stage | Key Findings / Evidence |
| --- | --- | --- | --- | --- | --- |
| Candida albicans | NDV-3A | N‑terminus of Als3 | Recombinant subunit vaccine | Clinical Phase II | Significantly reduced recurrence in patients with recurrent vulvovaginal candidiasis; cross-protection against multidrug-resistant C. auris in murine models. |
| Cryptococcus neoformans | Δsgl1 whole‑cell vaccine | Whole attenuated strain | Heat‑killed, chitosan‑deficient whole‑cell vaccine | Preclinical (mouse model) | Elicits robust protection in immunocompromised murine models; induces γδ T‑cell‑mediated immunity via TLR2 signaling. |
| Aspergillus fumigatus | Recombinant Asp f 3 + Asp f 9 liposomal vaccine | Asp f 3, Asp f 9 | Liposome‑encapsulated subunit vaccine | Preclinical (mouse model) | Protects against invasive pulmonary aspergillosis; enhances Th1‑type immune responses. |
| Multiple fungal pathogens | Glucan particle‑based vaccine system | Various recombinant antigens | β‑glucan particle delivery platform for antigen display | Preclinical (mouse model) | Enables targeted antigen delivery to immune cells; elicits protective responses in immunocompromised hosts against cryptococcosis and other IFIs. |
