# Supplementary material for: Immune evasion, dysregulation, and emerging immunotherapies for invasive fungal infections in the immunocompromised host
Source: Front Immunol. 2026 Apr 1;17:1788786. doi: 10.3389/fimmu.2026.1788786 (PMC13079191; doi:10.3389/fimmu.2026.1788786)
Supplement: Supplementary file 3 [file Table3.docx]

Supplementary Material 1: Detailed PubMed/MEDLINE Search Strategy

This supplementary material provides the complete and reproducible search strategy used in PubMed/MEDLINE for the narrative review entitled “Immune Evasion, Dysregulation, and Emerging Immunotherapies for Invasive Fungal Infections in the Immunocompromised Host.” The search was designed to capture relevant literature published between January 1, 2015, and December 31, 2025.

1. Search Date

Final search execution: December 15, 2025.

2. Database

PubMed/MEDLINE (via NCBI).

3. Search Query

The following combination of Medical Subject Headings (MeSH) and free-text terms was used, connected with Boolean operators (AND, OR). The search was not restricted by language at the time of searching, but only English articles were included during screening.

(

("Invasive Fungal Infections"[Mesh] OR "Mycoses"[Mesh] OR "Candidiasis, Invasive"[Mesh] OR "Aspergillosis"[Mesh] OR "Cryptococcosis"[Mesh] OR "Mucormycosis"[Mesh] OR "invasive fungal infection*"[tiab] OR "invasive mycosis"[tiab] OR "invasive candidiasis"[tiab] OR "invasive aspergillosis"[tiab] OR "invasive cryptococcosis"[tiab])

AND

("Immunocompromised Host"[Mesh] OR "Immunosuppression"[Mesh] OR "Neutropenia"[Mesh] OR "HIV"[Mesh] OR "Transplant Recipients"[Mesh] OR "Hematopoietic Stem Cell Transplantation"[Mesh] OR "immunocompromis*"[tiab] OR "immunosuppress*"[tiab] OR "immunodeficien*"[tiab] OR "neutropeni*"[tiab] OR "transplant*"[tiab] OR "HIV"[tiab] OR "AIDS"[tiab])

AND

("Immune Evasion"[Mesh] OR "Immune Tolerance"[Mesh] OR "Antigenic Variation"[Mesh] OR "Biofilms"[Mesh] OR "immune evasion"[tiab] OR "immune suppression"[tiab] OR "immune dysregulation"[tiab] OR "immunomodulat*"[tiab] OR "biofilm formation"[tiab] OR "virulence factor*"[tiab] OR "pathogen-associated molecular patterns"[tiab])

AND

("Immunotherapy"[Mesh] OR "Immune Checkpoint Inhibitors"[Mesh] OR "Adaptive Immunotherapy"[Mesh] OR "Vaccines"[Mesh] OR "Antibodies, Monoclonal"[Mesh] OR "Cytokines"[Mesh] OR "CAR-T cells"[tiab] OR "checkpoint inhibitor*"[tiab] OR "PD-1"[tiab] OR "PD-L1"[tiab] OR "vaccine"[tiab] OR "monoclonal antibody"[tiab] OR "cytokine therapy"[tiab] OR "adoptive cell therapy"[tiab])

)

AND

("2015/01/01"[Date - Publication] : "2025/12/31"[Date - Publication])

4. Search Notes

- Truncation symbols (*) were used to capture plural forms and related terms.

- Titles and abstracts (`[tiab]`) were searched alongside MeSH terms to increase sensitivity.

- The search was limited to the date range 2015–2025 to focus on recent advances.

- No filters for article type were applied during the initial search; screening was performed during study selection.

5. Search Results

The initial search yielded 2,847 records (as of December 15, 2025). These records were exported to a reference management software (EndNote X20) for deduplication and subsequent screening.

6. Screening Process

As described in Section 2.2 of the main manuscript, titles/abstracts were screened against eligibility criteria, followed by full-text review. Studies not meeting inclusion criteria (e.g., focused on immunocompetent hosts, lacking mechanistic or therapeutic insights, or published outside the date range) were excluded.

7. Manual Search Supplementation

To ensure comprehensive coverage, reference lists of included articles and relevant systematic reviews were hand-searched for additional eligible publications not captured by the electronic search.

This search strategy was designed to balance sensitivity and specificity and is reported in accordance with best practices for narrative reviews to ensure transparency and reproducibility.
